# Geospatial analyses identify regional hot spots of diffuse gastric cancer in rural Central America

**DOI:** 10.1186/s12885-019-5726-x

**Published:** 2019-06-07

**Authors:** Ricardo L. Dominguez, Charlotte B. Cherry, Dago Estevez-Ordonez, Robertino Mera, Veronica Escamilla, Michael Pawlita, Tim Waterboer, Keith T. Wilson, Richard M. Peek, Gloria Tavera, Scott M. Williams, Margaret L. Gulley, Michael Emch, Douglas R. Morgan

**Affiliations:** 1 Hospital de Occidente, Santa Rosa de Copan, Copan, Honduras; 2 Office of Public Health Informatics & Analytics, Tennessee Department of Public Health, Nashville, TN USA; 3 Vanderbilt Ingram Cancer Center (VICC), Vanderbilt University Medical Center, Nashville, USA; 4 Division of Gastroenterology, Hepatology, and Nutrition, Vanderbilt University Medical Center, Nashville, USA; 5 Carolina Population Center, University of North Carolina, Chapel Hill, USA; 6 Division of Molecular Diagnostics of Oncogenic Infections, German Cancer Research Center (DKFZ), Heidelberg, Germany; 7 Department of Population and Quantitative Health Sciences and Institute of Computational Biology, Case Western Reserve University, Cleveland, USA; 8 Department of Pathology, University of North Carolina, Chapel Hill, USA; 9 Department of Geography, University of North Carolina, Chapel Hill, USA; 10 Division of Gastroenterology and Hepatology, The University of Alabama at Birmingham (UAB), 1808 7th Avenue South, BDB 373, Birmingham, AL 35233 USA

**Keywords:** Gastric cancer, Diffuse gastric cancer, Germline mutations, *H. pylori*, Central America, CA-4, Honduras

## Abstract

**Background:**

Geospatial technology has facilitated the discovery of disease distributions and etiology and helped target prevention programs. Globally, gastric cancer is the leading infection-associated cancer, and third leading cause of cancer mortality worldwide, with marked geographic variation. Central and South America have a significant burden, particularly in the mountainous regions. In the context of an ongoing population-based case-control study in Central America, our aim was to examine the spatial epidemiology of gastric cancer subtypes and *H. pylori* virulence factors.

**Methods:**

Patients diagnosed with gastric cancer from 2002 to 2013 in western Honduras were identified in the prospective gastric cancer registry at the principal district hospital. Diagnosis was based on endoscopy and confirmatory histopathology. Geospatial methods were applied using the ArcGIS v10.3.1 and SaTScan v9.4.2 platforms to examine regional distributions of the gastric cancer histologic subtypes (Lauren classification), and the *H. pylori* CagA virulence factor. Getis-Ord-Gi hot spot and Discrete Poisson SaTScan statistics, respectively, were used to explore spatial clustering at the village level (30–50 rural households), with standardization by each village’s population. *H. pylori* and CagA serologic status was determined using the novel *H. pylori* multiplex assay (DKFZ, Germany).

**Results:**

Three hundred seventy-eight incident cases met the inclusion criteria (mean age 63.7, male 66.3%). Areas of higher gastric cancer incidence were identified. Significant spatial clustering of diffuse histology adenocarcinoma was revealed both by the Getis-Ord-GI* hot spot analysis (*P*-value < 0.0015; range 0.00003–0.0014; 99%CI), and by the SaTScan statistic (*P-*value < 0.006; range 0.0026–0.0054). The intestinal subtype was randomly distributed. *H. pylori* CagA had significant spatial clustering only in association with the diffuse histology cancer hot spot (Getis-Ord-Gi* *P* value ≤0.001; range 0.0001–0.0010; SaTScan statistic P value 0.0085). In the diffuse gastric cancer hot spot, the lowest age quartile range was 21–46 years, significantly lower than the intestinal cancers (*P* = 0.024).

**Conclusions:**

Geospatial methods have identified a significant cluster of incident diffuse type adenocarcinoma cases in rural Central America, suggest of a germline genetic association. Further genomic and geospatial analyses to identify potential spatial patterns of genetic, bacterial, and environmental risk factors may be informative.

## Background

Gastric adenocarcinoma is the leading global cause of infection-related cancer mortality and overall is the third leading cause of cancer death [1–4]. Approximately 1 million incident cases are projected annually, with the majority of incident cases observed in eastern Asia, Latin America, and eastern Europe. Nearly 70% of global cancers now occur in low/middle income countries (LMICs), and seven cancers, including gastric cancer, account for 70% of the LMIC cancer mortality burden [2].

Gastric cancer has marked geographic variability, assessed at the regional, country, and within-country levels [5]. In Latin America, a significant burden of disease is concentrated in the mountainous regions along the Pacific littoral [6, 7], the gastric cancer “altitude enigma”, and may represent host genetic variation*. Helicobacter pylori (H. pylori)* virulence factors, and dietary and environmental risk exposures also play a role in the rural mountain villages [6]. There is also evidence that disrupted host-*H. pylori* coevolution, with mismatch of respective genetic ancestries, may play a role in cancer predisposition in Latin America [8–10]. *H. pylori* is the most common chronic bacterial infection in the world, affecting half of the world’s population. Infection prevalence ranges from 20 to 35% in high income countries to 60–90% in LMICs [10–12]. *H. pylori* CagA has been shown to be an important virulence factor for disease progression to gastric adenocarcinoma [13–16]. The principal subsets of gastric adenocarcinoma per the Lauren histologic classification are intestinal and diffuse, and recent findings in The Cancer Genome Atlas (TCGA) NIH initiative confirm these subtypes [17–19].

Germline genetic associations may be present in 5–10% of gastric adenocarcinoma [20]. Hereditary diffuse gastric cancer (HDGC) is uncommon, and primarily driven by *CDH1* mutations [20]. Recent studies suggest that homologous recombination (HR) germline mutations (*PALB2, BRCA1, RAD51C*) are also important in familial clusters, including in Latin America [21]. Some familial clustering may also be attributable to shared bacterial or environmental exposures. Globally, intestinal gastric cancer is more common than diffuse by a 4.6:2 ratio [19], although in Central America and in Hispanics in the U.S., the diffuse subtype has a higher prevalence [22].

Geospatial methods and the use of geographic information systems (GIS) can delineate disease distributions and etiology, as well as inform prevention programs, however, few studies have applied spatial techniques to examine gastric cancer [23–26]. The objective of this study was to utilize geospatial methods to examine the spatial distributions of gastric cancer subtypes, in the context of an ongoing population-based, case-control study in Central America. The identified clusters of high gastric cancer incidence may implicate germline genetic associations, along with bacterial, dietary, or environmental co-factors.

## Methods

### Study design and setting

We performed spatial cluster analyses in the context of an active population-based, case-control study centered in western Honduras. The study was set in rural Honduras and is representative of the Central America Four (“CA-4”) region (Guatemala, Honduras, El Salvador, Nicaragua), the largest LMIC region in the western hemisphere, with over 36 million inhabitants [26]. This mountainous region has a racial-ethnic mixture of primarily Hispanic Mestizo (95%) and has among the highest gastric cancer incidence rates in the western hemisphere, with a high prevalence of *H. pylori* infection of over 80% [2, 27–29].

We prospectively identified all incident cases of gastric cancer between 2002 and 2013 from a registry within the Ministry of Health district hospital (Hospital de Occidente) of western Honduras in Santa Rosa de Copán, that serves as the principal referral center for the region. The hospital catchment area has been previously described [28]. The diagnosis of gastric cancer was based on endoscopic appearance and confirmatory histopathology. Western Honduras includes all or part of the three western states (*departamentos*) of Honduras, spanning 5000 km^2^, with an adult population of approximately 400,000. Each state is comprised of counties (municipalities) consisting of villages (aldeas) of 30–50 households on average, each with a unique geocode. Incident cases in the villages of the three western states in the referral area were included in the analysis. The crude incidence rates for each village were calculated for GIS mapping**:** number of cases per village population per 100,000 persons for the overall study period. The population data for each village was obtained from the Honduras Census Institution (Instituto Nacional de Estadistica) from the census year 2001 [30].

### *H. pylori* infection assessment

*H. pylori* and CagA status were determined by the novel validated *H. pylori* multiplex serology. This antibody detection technology uses 15 *H. pylori* proteins bacterially expressed in full length as recombinant proteins in fusion with N-terminal glutathione-S-transferase (GST) and C-terminal a small tagging epitope (tag). Each GST-X-tag fusion protein was bound and affinity-purified on a different bead set with glutathione surface and marked with a distinct internal fluorescent color (SeroMap, Luminex Corp., Austin, TX, USA) [31–36]. Seropositivity against each of the 15 *H.pylori* antigens, including CagA, was defined based on antigen-specific cut-point values, previously determined in validation studies [29]. Positive *H. pylori* status was defined as seropositivity against more than 3 of these antigens.

### Geospatial and statistical analysis

The Getis Ord Gi* local spatial cluster analysis method was implemented in ArcGIS® version 10.3.1 (ESRI, Redlands, CA, USA) [37]. This method analyzes each feature in the context of its neighboring features (e.g., villages). A village (aldea) with a high value surrounded by other villages with high values may be a statistically significant hot spot. A fixed distance band of 23 km (~ 14 miles) was used to examine spatial relationships of aldeas. We selected a 23 km distance to ensure that each aldea had multiple neighbors. The local sum of cases for each aldea and its neighbors is compared proportionally to the sum of all cases in the study area. If the local sum is very different from the expected local sum (and is too large to be random) it is a statistically significant hot (or cold spot). Cluster *P* values were adjusted for multiple testing using the False Discovery Rate (FDR) correction. Kulldorff’s spatial scan statistic, a complementary methology, was used to validate the hot spot analysis results using SaTScan 9.4 (Boston, MA) [38]. A discrete Poisson model was used to identify high gastric cancer incidence clusters. The spatial scan statistic uses a circular roving window varying in size, that increases incrementally to encompass a maximum percent of the population (25, 50%, etc.). We set the maximum window size to encompass up to 25% of the population. Counts that are higher than expected relative to the underlying population and study area are designated as a cluster. Cluster significance was determined using Monte Carlo simulation (999 permutations).

Diffuse histology cancer cases and intestinal histology cancer cases were analyzed separately. Cases of mixed or indeterminate histology were excluded from the geospatial analysis. The *H. pylori* CagA spatial distribution was analyzed subsequently. Gastric cancer cases without aldea geocodes and aldea population data, were excluded from the geospatial analysis, by necessity, but included in the overall descriptive analyses of the study population. Demographic and clinical data were analyzed by chi-squared and univariable multinomial logistic regression analyses.

## Results

A total of 702 gastric cancer patients were identified in the western Honduras gastric cancer registry from 2002 to 2013. There were 498 subjects with either intestinal or diffuse subtypes, with a mean age of 63.2 (SD 13.8), and 66.3% males (*n* = 330). Three hundred seventy-eight patients had validated village-level (aldea) geocode data for the spatial analysis. (Table 1). The subjects without village-level geocodes were primarily from the early study period, which primarily focused on county-level geocoding. The excluded subjects (*n* = 204) were from outside of the district hospital catchment area (*n* = 108), with non- intestinal/diffuse histology (*n* = 80; mixed 42; indeterminate 35; other 3), or had missing data (*n* = 16).

**Table 1:** Demographic and exposure factors of the intestinal and diffuse type gastric cancer cases

Characteristics	Overall population	Spatial analysis cases	Cases without village geocodes^b^	*P* value
Cancer Cases (N)	498	378	120	0.047
Intestinal subtype	259 (52.0%)	187 (49.5)	72 (60)	
Diffuse subtype	239 (48.0%)	191 (50.5)	48 (40)	
Age, mean (SD)	63.2 (13.8)	62.6 (13.9)	65.4 (13.6)	0.054
Gender				0.51
Female (%)	168 (33.7)	131 (34.7)	37 (30.8)	
Male (%)	330 (66.3)	247 (65.3)	83 (69.2)	
Family history GC (%)				0.81
Yes (%)	33 (6.6)	26 (6.9)	7 (5.8)	
No (%)	448 (90.0)	340 (89.9)	108 (90.0)	
Not reported (%)	17 (3.4)	12 (3.2)	5 (4.2)	
Alcohol history (ever)				0.72
Yes (%)	107 (21.8)	79 (21.4)	28 (23.3)	
No (%)	373 (76.1)	284 (76.8)	89 (74.2)	
Not reported (%)	18 (3.6)	15 (4.0)	3 (2.5)	
Smoking history (ever)				0.72
Yes (%)	126 (24.1)	97 (26.6)	29 (24.8)	
No (%)	355 (73.6)	267 (73.2)	88 (75.2)	
Not reported (%)	17 (3.4)	14 (3.7)	3 (2.5)	
*H. pylori* serodiagnosis^a^	385	286	99	0.82
Positive N (%)	337 (87.5)	251 (87.8)	86 (86.9)	
Negative N (%)	48 (12.5)	35 (12.2)	13 (13.1)	
*H. pylori* CagA antibodies^a^	385	286	99	0.64
Positive N (%)	361 (93.8)	267 (93.4)	94 (95.0)	
Negative N (%)	24 (6.2)	19 (6.6)	5 (5.0)	

^a^The comparison *P* values refer to the spatial analysis cases with geocodes versus the excluded cases without the village-level (aldea) geocodes. ^b^In the initial study period, geocodes were at times limited to the municipality-level, without village-level data

^a^*H. pylori* and CagA multiplex assay data were not available for all subjects in the study populations

In the overall study population, there were 259 cases (52%) of the intestinal subtype. Over three quarters of cancer patients (87.5%) were positive for *H. pylori* infection by multiplex serology, of whom 93.8% were CagA positive. In this setting, tobacco and alcohol use was limited, with proportions of never-used of 73.6 and 76.1%, respectively. A family history of gastric cancer was only noted in 6.6% of cases. No significant differences in demographic or clinical features were noted between the geospatial analysis study group (with aldea geocodes) and the subject group without geocodes, with the exception of borderline differences (*P* = 0.05) in age and proportions of the Lauren classification subtypes.

**Fig. 1:**
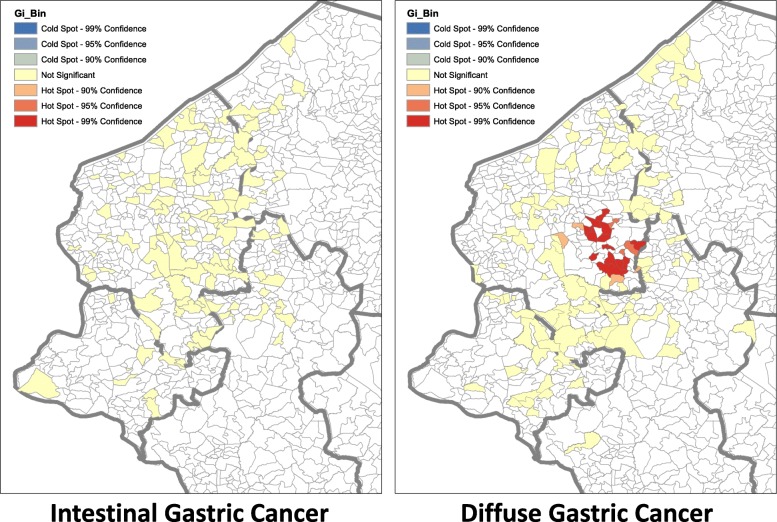
Spatial clustering of diffuse type gastric cancer, Getis Ord Gi* hot spot cluster analysis. Spatial clustering of diffuse gastric cancer incident cases were identified by two independent GIS methodologies. The Getis Ord Gi* hot spot analysis (Fig. 1), identified three neighboring hotspots in western Honduras that may be considered one cluster area (*P* value < 0.0015; range 0.00003–0.0014; 99% CI). The spatial scan statistic SaTScan (not shown) also demonstrated a statistically significant cluster (32 km radius) in the same location (*P*-value < 0.006; range 0.0026–0.0054). The intestinal subtype cancers were randomly distributed, and without high incidence clusters

In the study period, in the catchment area, incident cases were identified in 219 villages within a total of 58 counties. Hot spots of diffuse gastric cancer incidence were identified by both independent methodologies (Fig. 1). The Getis Ord Gi* hot spot analysis identified three neighboring hotspots in western Honduras (*P* value < 0.0015; range 0.00003–0.0014; 99% CI) that may be considered one cluster area. The spatial scan statistic results corroborated the hotspot findings. A single statistically significant cluster with a 32 km radius was identified in the same location as the hotspots using SaTScan (*P*-value < 0.006; range 0.0026–0.0054). The intestinal subtype cancers were randomly distributed, and without high incidence clusters. (Fig. 1).

**Fig. 2:**
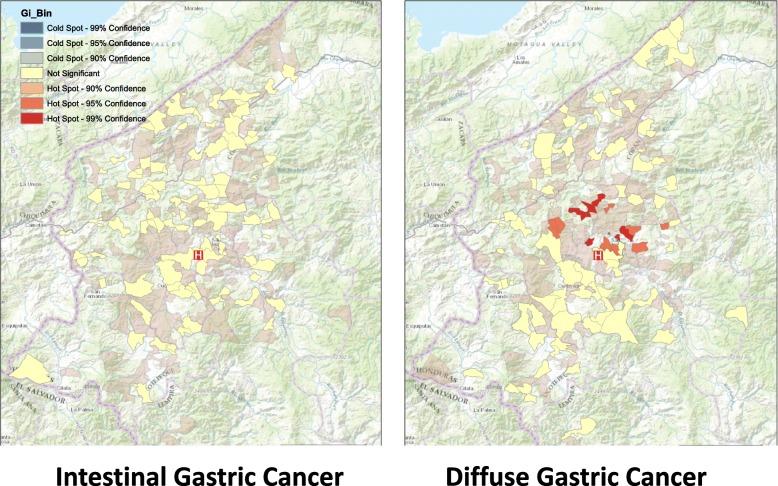
Spatial analysis of *H. pylori* CagA, by Getis Ord Gi* hot spot cluster analysis. Clusters with a higher relative number of cases with *H. pylori* CagA infection were identified, but only in association with the incident diffuse cancer clusters. CagA hotspots were detected using the Getis-Ord Gi* statistic (*P* value ≤0.001; range 0.0001–0.0010), as shown in Fig. 2. The *H. pylori* CagA hotspots was also observed with the SaTScan statistic (*P*-value < 0.0085) in the same area (not shown). This indicates that CagA may be a co-factor in the diffuse gastric cancer cluster area. CagA was randomly distributed among the diffuse cancers outside of the hotspot. CagA was also randomly distributed among the intestinal cancers in the hotspot area and in the western Honduras as a whole

Clusters of aldeas with a higher relative number of cases of *H. pylori* CagA were identified, but only among the diffuse type cancer hotspot clusters. Hotspots were detected using the Getis-Ord Gi* statistic (*P* value ≤0.001; range 0.0001–0.0010). (Fig. 2). The location of the *H. pylori* CagA hotspots was also observed with the SaTScan statistic, with a significant cluster (*P*-value < 0.0085) with a 4 km radius, located in the same area. The overlap of the clustering areas of diffuse cancer and CagA areas was noted, indicating that CagA may be a co-factor in the area where germline mutations may be important. CagA was randomly distributed outside of the diffuse cancer cluster hotspot, and also among the intestinal cancers within this hotspot area.

We examined differences in demographic and exposure factors between cases that were located in the diffuse hotspot versus cases that were intestinal as well as all cancers outside of the cluster area. Table 2 summarizes the demographic and exposure factors of the hot spot (cluster) of diffuse gastric cancer for the 378 patients for which village-level geocodes were available. *H. pylori* multiplex serology data was available for 286 out of the 378 cases in the geospatial analysis. Importantly, diffuse cases in the diffuse gastric cancer hot spot had the lowest age interquartile 0–25% range, 21–46 years, and were significantly younger than the intestinal cases located within the diffuse cluster (*P* = 0.024). The modest number of cases within the diffuse cancer hot spot may have limited the power to detect some significant differences in other risk exposures.

**Table 2:** Demographic and exposure factors of the hot spot (cluster) of diffuse gastric cancer cases

Characteristics	Cluster, Diffuse	Cluster, Intestinal	Non-cluster, Diffuse	Non-cluster, Intestinal
Geospatial methods^a^, *P*-values • Getis Ord Gi • SaTScan	*P* < 0.0015,99%CI *P* < 0.006	Referent	Referent	Referent
Cases (N)	52	32	139	155
Histology, signet ring	75%	na	82%	na
Age, mean (SD)	60.2 (16.5)	69.7 (9.9)	61.3 (14)	63 (13.1)
Age IQR	46–73	62–76	52–72	53–73
Age IQR 0–25%	21–46	53–62	23–52	30–53
*P*-values	Referent	*p* = 0.002	*p* = 0.61	*p* = 0.034
Gender				
Female N (%)	15 (28.9)	11 (34.4)	50 (36.0)	55 (35.5)
Male N (%)	37 (71.1)	21 (65.6)	89 (64.0)	100 (64.5)
*P*-values	Referent	*p* = 0.59	*p* = 0.36	*p* = 0.38
Family history^a^(N)	51	28	136	151
Yes (%)	2 (3.9)	3 (10.7)	7 (5.2)	14 (9.3)
No (%)	49 (96.1)	25 (89.3)	129 (94.9)	137 (90.7)
*P*-values	Referent	*p* = 0.25	*p* = 0.73	*p* = 0.24
Alcohol history, ever (N)	50	31	134	148
Yes (%)	13 (26)	10 (32.3)	25 (18.7)	31 (21)
No (%)	37 (74)	21 (67.7)	109 (81.3)	117 (79)
P-values	Referent	*p* = 0.54	*p* = 0.28	*p* = 0.46
Smoking history, ever (N)	50	31	134	149
Yes (%)	13 (26)	13 (42)	34 (25.4)	37 (24.8)
No (%)	37 (74)	18 (58)	100 (74.6)	112 (75.2)
*P*-values	Referent	*p* = 0.14	*p* = 0.93	*p* = 0.87
*H. pylori* serodiagnosis^a^ (N)	38	25	107	116
Positive N (%)	31 (81.6)	20 (80)	96 (89.7)	104 (89.7)
Negative N (%)	7 (18.4)	5 (20)	11 (10.3)	12 (10.3)
*P*-values	Referent	*p* = 0.88	*p* = 0.20	*p* = 0.195
*H. pylori* Cag A antibodies^a^ (N)	38	25	107	116
Positive (%)	33 (86.8)	22 (88)	100 (93.5)	112 (96.5)
Negative (%)	5 (13.2)	3 (12)	7 (6.5)	4 (3.5)
*P*-values	Referent	*p* = 0.89	*p* = 0.21	*p* = 0.039

^a^Table 2 summarizes the demographic and exposure factors of the hot spot (cluster) of diffuse gastric cancer for the 378 patients for which village-level geocodes were available. In the initial study period, geocodes often limited to the municipality-level, without village-level data. *H. pylori* CagA multiplex serology data was available for 286 out of the 378 cases in geospatial analysis

^a^The cluster detection methods identify areas with high prevalence villages adjacent to other high prevalence villages. Therefore, while the prevalence is higher outside of the cluster, those patterns of higher incidence appear to be random

## Discussion

Gastric cancer, the leading infection-associated cancer, demonstrates remarkable geographic variability. In Latin America, the burden is concentrated in the Pacific littoral mountainous regions of Mexico, Central America, and the Andes [6]. We have identified hot spots of the diffuse gastric cancer subtype, in the mountainous region of western Honduras, with rigorous geospatial methods. We postulate that the geographic clustering and younger age of the diffuse gastric cancer patients may implicate a germline genetic association. This may represent a cluster of subjects with germline mutations of *CDH1,* another tumor suppressor gene, or other recently described associations [20, 39–42].

The cluster of diffuse adenocarcinomas may be due to several factors, including germline mutations, and may represent a Founder and/or endogamy effects. Kaurah et.al., observed a combination of both a Founder effect and endogamy influences in hereditary diffuse gastric cancer (HDGC) in rural British Colombia [43–45]. While all of the Central American populations originally derived from Asian migrations, it is possible that they were founded by different sub-groups, a migration Founder effect. This could be tested using large-scale genomic data. Similarly, following Spanish colonization there were substantial bottlenecks across Central America with high mortality due to newly introduced infections (eg. smallpox), a potential extinction Founder effect. The patterns of diffuse cancer may be affected by varying degrees of host bottlenecks across our sites. Lastly, endogamy may affect genetic risk in the isolated mountain villages, either due to mating patterns, patterns or the small effective population sizes. In the latter case, we would predict that the clusters would have smaller village population sizes.

The familial clustering in 5–10% of gastric cancer cases may be partially attributed to shared *H. pylori* virulence factors, as well as environmental, dietary, and behavioral factors [20]. Specific *H. pylori* virulence factors may cluster in families and populations. The CagA clusters in our analysis are directly linked with the diffuse gastric cancer cluster. This is likely a secondary association and co-factor with the postulated germline genetic association, given the lower overall CagA prevalence within the cluster area, as well as the younger age of the diffuse cancer patients. It could also be postulated that patterns of host and pathogen genetic variation differ by region, thereby disrupting a co-evolutionary history as we have previously demonstrated in Colombia [8]. Environmental factors may be important in some areas. For instance, volcanic soils have been proposed as a contributory factor in the Middle East [24]. Lastly, EBV infection accounts for 10% of global gastric cancer [19]. In Honduras, we have noted a prevalence of 9%, without apparent geographic variation [46]. Polymorphisms in the EBV viral genome may impact oncogenicity, and warrant investigation of potential spatial patterns. Some EBV genome variants encode epitopes affecting innate or adaptive T cell response, implying that virulence factors may alter normal mechanisms controlling virus-induced cell growth [47, 48]. In sum, interactions between *H. pylori*, EBV and other components of the microbiome may also play a role in regional variation [49].

The principal limitations in the study are those inherent in spatial epidemiology. The finding of geographic hot spots of diffuse gastric cancers or *H. pylori* virulence factors may represent statistical artifacts albeit unlikely given the highly significant levels by two distinct methodologies. By necessity for the spatial analysis, cases outside of the western Honduras catchment areas and without aldea geocodes may have limited the sample size somewhat, and the ability to detect environmental associations. Exclusion of these populations would be unlikely to affect the principal spatial analysis in the defined catchment area.

## Conclusion

Geospatial methods have identified a significant cluster of diffuse gastric adenocarcinoma patients, in a high incidence region of Central America. Investigation of potential germline mutations in this cluster of diffuse cancers is warranted. Further analyses to also study potential spatial patterns of bacterial and environmental risk factors may also be insightful.

## Abbreviations

CA-4: Central America Four region: Honduras, Guatemala, Nicaragua, and El Salvador
EBV: Epstein-Barr virus
GIS: Geographic information systems
HDGC: Hereditary diffuse gastric cancer
LMIC: Low/middle income country
TCGA: The Cancer Genome Atlas
